# Why Do African Elephants (*Loxodonta africana*) Simulate Oestrus? An Analysis of Longitudinal Data

**DOI:** 10.1371/journal.pone.0010052

**Published:** 2010-04-07

**Authors:** Lucy A. Bates, Rosie Handford, Phyllis C. Lee, Norah Njiraini, Joyce H. Poole, Katito Sayialel, Soila Sayialel, Cynthia J. Moss, Richard W. Byrne

**Affiliations:** 1 School of Psychology, University of St Andrews, St Andrews, United Kingdom; 2 Department of Psychology, University of Stirling, Stirling, United Kingdom; 3 Amboseli Trust for Elephants, Nairobi, Kenya; Royal Holloway University of London, United Kingdom

## Abstract

Female African elephants signal oestrus via chemicals in their urine, but they also exhibit characteristic changes to their posture, gait and behaviour when sexually receptive. Free-ranging females visually signal receptivity by holding their heads and tails high, walking with an exaggerated gait, and displaying increased tactile behaviour towards males. Parous females occasionally exhibit these visual signals at times when they are thought not to be cycling and without attracting interest from musth males. Using demographic and behavioural records spanning a continuous 28-year period, we investigated the occurrence of this “simulated” oestrus behaviour. We show that parous females in the Amboseli elephant population do simulate receptive oestrus behaviours, and this false oestrus occurs disproportionately in the presence of naïve female kin who are observed coming into oestrus for the first time. We compare several alternative hypotheses for the occurrence of this simulation: 1) false oestrus has no functional purpose (e.g., it merely results from abnormal hormonal changes); 2) false oestrus increases the reproductive success of the simulating female, by inducing sexual receptivity; and 3) false oestrus increases the inclusive fitness of the simulating female, either by increasing the access of related females to suitable males, or by encouraging appropriate oestrus behaviours from female relatives who are not responding correctly to males. Although the observed data do not fully conform to the predictions of any of these hypotheses, we rule out the first two, and tentatively suggest that parous females most likely exhibit false oestrus behaviours in order to demonstrate to naïve relatives at whom to direct their behaviour.

## Introduction

Many species display visual, auditory and behavioural cues that indicate sexual receptivity, in addition to chemical signals of reproductive status [1]. These behavioural cues may be genetically determined and developmentally programmed, [2], or controlled and displayed through proceptive behaviour [3]. Male and female African and Asian elephants use chemical indicators of sexual status in order to coordinate sexual encounters [4]–[6]. Mature males (>25 years) undergo an annual period of sexual activity, known as musth, during which they experience heightened testosterone levels, continuously dribble large quantities of strongly scented urine, become dominant to all other non-musth males, and are the preferred mates of females [4], [7]–[10]. Females advertise imminent ovulations by chemicals released in urine [5], [11]. Female African elephants also display visual signals of their receptivity, specifically changes to their posture and gait and increased tactile behaviours, during their week-long ovulatory periods [12]–[14].

Musth is an honest signal of fitness in male elephants, so it is important for the reproductive success of female elephants that they direct their oestrus behaviours towards musth males [8], [12]. These older, larger, and stronger musth-males are also able to provide respite for receptive females who would otherwise be chased and continuously harassed by the advances of a series of younger males, so females enter into consort with them, sometimes for several days [8], [12]. This strategy of mate choice and consort behaviour is apparently acquired gradually by females. While older, experienced females (>25 years) consort with the highest-ranking musth males and actively avoid other males by running away from them, young females typically do not direct their oestrus behaviours appropriately when they first come into oestrus, and are more likely to run away from the larger musth males. We speculate that young females avoid musth-males because of their large size, which can be up to twice the body weight of an adolescent nulliparous female, but this idea has not yet been tested empirically (see supporting information files: Document S1 and Table S1). Young females are thus frequently chased and mounted by a succession of young- and non-musth males [8], [12]. As females mature, their behaviour changes to avoiding young males, and accepting the large musth-males. Acquisition of oestrus behaviours typical of mature females is thought to be based on individual experience, facilitated by observation of older females in the family group [15].

There are reports from a long-term study of wild African elephants that mature females occasionally ‘simulate’ the visual and behavioural signals of oestrus at times when they are not likely to be ovulating, but that coincide with the first occasions that young, nulliparous (never given birth) female relatives come into oestrus [15]. Mature females can be observed to approach and avoid males, run with their young oestrus relative during chases, and occasionally make post-copulatory calls after the young female is mated.Here we examine the occurrence of these simulated or false oestrus events using the unique database of wild elephant behaviour collected from Amboseli National Park, Kenya [16]. We aim to determine when parous female elephants are most likely to exhibit false oestrus, and compare the pattern of false oestrus events against predictions made by several alternative hypotheses, in an attempt to establish the most likely explanation for the occurrence of false oestrus in African elephants. The hypotheses we shall consider are: 1) false oestrus has no functional purpose; 2) false oestrus increases the reproductive success of the simulating female; and 3) false oestrus increases the inclusive fitness of the simulating female.

## Methods

### Study Site and Population

Since 1972, the Amboseli Elephant Research Project (AERP), part of the Amboseli Trust for Elephants [16], has identified and named over 2200 elephants living within or around the Amboseli National Park, Kenya. The AERP has recorded details of all births and deaths occurring in the Amboseli elephant population during this time. By December 2006 the population was known to include 1400 living elephants, divided into 58 family units of adult females and their dependent calves, with approximately 300 independent adult males.

### Data Collection

The long-term researchers, Cynthia Moss, Joyce Poole, and Phyllis Lee, and permanent research staff, Norah Njirani, Soila Sayialel, and Katito Sayialel, provided the bulk of behavioural data collection for this study, totalling approximately 480 months of daily elephant observations. Elephants are encountered on an ad libitum basis. All researchers then employ focal behaviour sampling to record details of every presumed oestrus event they observe, as determined by postural and behavioural changes in the female, and by interest and pursuit from adult males after smelling her urine and vulva. The observers note which females are displaying oestrus behaviour, which males are present and following or guarding the female, the musth status of all males present, and whether a sexual interaction occurs. All oestrus events observed are subsequently recorded in a dedicated Microsoft Access database. The researchers may also write longhand field notes on these observations, recording ad libitum any unusual behaviours or interactions.

### Data Mining

The oestrus database comprises 999 oestrus events of identified females, observed between 1976 and 2004. We extracted all occasions from the oestrus database when two or more females from the same family group were recorded as showing behaviour characteristic of oestrus at the same time (that is, within seven days of each other, because the receptive period of the female cycle lasts for approximately one week, within an oestrus cycle of 16 weeks [5]). We then cross-referenced the lists of single (901 events) and coincident (98) oestrus events with the demographic records to determine which oestrus events must have been false. Oestrus events were classified according to the criteria detailed below.

We recorded the oestrus event of a parous female as ‘false’ if the female was pregnant, senescent, or in a state of lactation induced infertility. Pregnancy was determined by a birth less than 22 months after the observed oestrus event. Senescence applied to females over the age of 50 that had not given birth to any calves for a period of at least 5 years before the observed oestrus behaviour, and with no further calves subsequently born. Lactation induced infertility referred to females that had given birth to a surviving calf 4 months or less before the observed oestrus event. Thus, we recorded the oestrus event as ‘genuine’ if it was observed more than 4 months after the last birth; 4 months is derived from the minimum known inter-birth interval (IBI) between successive surviving calves (26 months), minus the 22-month gestation period. This criterion is very conservative as the median duration to next conception if the previous calf survives is 31 months (equivalent to a 53 month IBI) [16]. If the last-born calf was dead, we presumed all oestrus events occurring after the death were genuine.

If a female was nulliparous, we treated all observed oestrus events as genuine, because we have no means of determining otherwise. Modal latency to first birth after the first observed oestrus event is 22 months, suggesting most nulliparous females do indeed conceive on their first observed cycle.

### Data Analysis

Having identified the cases of false oestrus, we used the demographic records, oestrus database and field notes collected by AERP researchers to test all proposed hypotheses.

All statistical analyses were carried out using SPSS v.12, with α = 0.05. All hypotheses tested were two-tailed. Due to the unequal sample sizes of the three nulliparous female group conditions (A: only female in family group exhibiting oestrus (n = 251); B: oestrus coincided with a genuine oestrus of a parous female relative (n = 11); C: oestrus coincided with a false oestrus event of a parous female relative (n = 10)), we used non-parametric independent-samples statistics. The chi-square goodness of fit test was used to explore the distribution of false oestrus events. The Kruskal-Wallis ANOVA was used to compare the number of males present, the copulations observed, and the birth latencies for nulliparous females in the three conditions. A 2*2 Fisher exact test was used to compare the mate choices of nulliparous females showing oestrus alone with those who coincided with a false oestrus, and also to explore the mate choices of parous females. A parametric independent samples t-test was used to compare the number of genuine oestrus events potentially observed between nulliparous females whose first oestrus coincided with a false oestrus, and nulliparous females from the same families who experienced their first oestrus event alone. The data for this analysis met with the assumptions of parametric analysis.

## Results

### Occurrence of false oestrus events

Of the 999 observed oestrus events of known females, all believed to be genuine by researchers when they were recorded, 2% (n = 19) were false, as determined by subsequent birth records. For 14 of these 19 false oestrus events, the female showing oestrus-like behaviour was already pregnant; on four occasions she was senescent; and on one occasion the female showing oestrus-like behaviour was in a state of lactation-induced infertility. The 19 false oestrus events were all observed in different individuals, from 12 different family groups.

**Figure 1:**
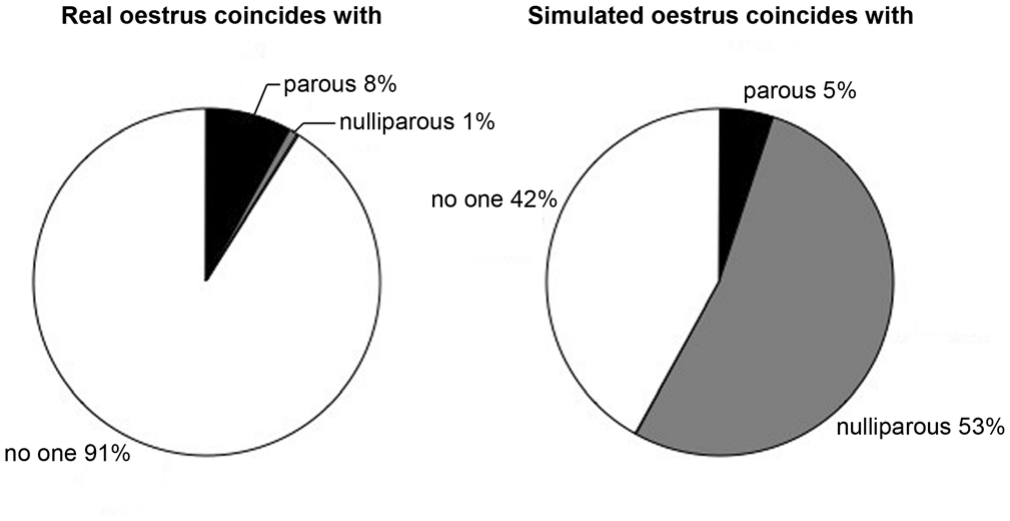
Coincident occurrence of real and false oestrus events. The first panel illustrates the pattern of occurrence of the 980 genuine oestrus events, and the second panel shows the 19 false oestrus events. Each panel shows how many events occurred singly in a family, how many coincided with a genuine oestrus event of another parous female, and how many with a nulliparous female.

Coincidence of genuine oestrus events was low: only 9% (n = 87) of genuine oestrus events coincided with an oestrus period of another female in the same family (76 with a parous relative, and 11 with a nulliparous relative, no individual contributed more than one data point). However, 58% of the 19 false oestrus events (n = 11) coincided with an observed, genuine oestrus period of another female in the same family (Table 1; Fig.1). These coincidences occurred 10 times with a nulliparous female and once with another parous female. At least four of the eight false oestrus events that did not coincide with any *observed* oestrus did occur in the same month as a nulliparous female relative conceived, as determined by the subsequent birth records. However, we have to treat these four cases as though they did not coincide with any other oestrus event because the exact date and details of the nulliparous females' oestrus period were not observed or recorded by the AERP. For the remaining four cases, there is no indication from subsequent birth records that the false oestrus event coincided with oestrus of any other member of the family. We cannot explain why false oestrus was observed in these four cases; only further data collection will indicate if they are anomalous cases or not.

**Table 1:** Occurrence of oestrus events coincident with kin.

	Real oestrus	False oestrus
Coincident with:		
No other elephant	893 (883.9)	8 (17.1)
Parous female	76 (75.5)	1 (1.5)
Nulliparous female	11 (20.6)	10 (0.4)

Chi square expected frequencies are shown in parentheses.

Of the 10 coincidences between nulliparous and parous females, the parous female was the mother of the nulliparous female in four cases, an older sister in two cases, and the matriarch of the family in four cases. The coincidence between the two parous females concerned a mother and daughter pairing. The expected number of coincidences of false oestrus events with the genuine oestrus of a nulliparous female was close to zero (Table 1). Coincidence of false oestrus with the oestrus events of nulliparous females was significantly greater than expected from the pattern of coincident oestrus events where both were genuine, whereas the observed frequencies of coincidence with another parous female are consistent with what is expected by chance (chi square test: χ^2^ =  240.37, df = 2, p<0.001, see Figure 1).

Why should false oestrus events occur with such unexpectedly high probability in the presence of young, nulliparous relatives? We shall now explore several alternative possibilities.

### No function

At the most trivial, false oestrus behaviour might not have any functional consequence but simply result from hormonal changes, perhaps as a result of pregnancy or through associating with females that are genuinely experiencing oestrus. However, the pattern of false oestrus events does not fit the predictions either of these possibilities. Firstly, five of the females showing false oestrus behaviours were not pregnant. For those that were (n = 14), there was little commonality as to when during a pregnancy false oestrus was observed: the median occurrence was at 15 months into the pregnancy, with a range of 2–19 months (mean 12 months, sd ±5). Thus false oestrus events could occur at any point during the 22-month gestation. Secondly, in at least four of the cases, there was no other female in the family in oestrus at the time of the simulation. Hormonal changes due to pregnancy or associating with genuinely receptive females therefore fail to explain the pattern of occurrence of false oestrus events, particularly the high rate of coincidence with nulliparous female relatives.

### Increasing own reproductive success

Might females simulate oestrus for direct gain? By simulating oestrus behaviour and thus receiving the attentions of males, females might induce their own sexual receptivity when a suitable male is available [17], increasing their reproductive success. This explanation seems unlikely, however, as the female was already pregnant in 14 of the 19 false oestrus events, and in four other cases the female was senescent and did not conceive any more offspring. Even for the female in lactational anoestrus, her inter-birth interval was 49 months, meaning that she did not successfully conceive until 27 months after the birth of her previous calf, some 23 months after exhibiting false oestrus behaviours. This delay falls well within the normal IBI range (median  = 53 mo, 75% range  = 48–62 mo) [16]. Again, the pattern of occurrence of false oestrus behaviour is not satisfactorily explained.

### Increasing inclusive fitness

Might the pattern of occurrence of false oestrus events be explained by the inclusive fitness benefit of enhancing the reproductive success of a related female who is genuinely receptive? As there is only one example of coincidence with a parous female, this case cannot be properly analysed, so we shall concentrate on the 10 cases that coincide with oestrus of a nulliparous female. The most obvious possible benefit to the females lies in sexual access. More males might be attracted to the family group, giving the genuinely receptive, nulliparous female increased mate choice and potentially increasing sperm competition, both of which can improve offspring viability [18]–[20]. Additionally, those musth males already associated with a family group might be retained for longer, thus increasing the time the genuinely receptive female has available for copulating.

To assess a nulliparous female's access to males, we counted the total number of males present with a family group, and the number of musth males present, depending on whether (A) she was the only female exhibiting oestrus (n = 251), (B) her oestrus coincided with a genuine oestrus of another female (n = 11), or (C) her oestrus coincided with a false oestrus of another female (n = 10). Neither estimate of access to males showed significant variation (median, min-max range; total males: A = 1, 0–14 males; B = 1, 0–3, C = 2, 0–5; Kruskal Wallis ANOVA: χ^2^ = 2.77, df = 2, p = 0.25; musth males only: A = 1, 0–4 males; B = 0, 0–2; C = 1, 0–4; χ^2^ = 3.48, df = 2, p = 0.18). To assess a nulliparous female's sexual activity, we counted the number of males with whom she was recorded copulating and her total number of copulations in the same three conditions. Neither measure of sexual activity showed significant variation (median, min-max range; males copulated: A = 0, 0–5 males, B = 0, 0–3; C = 0.5, 0–4; Kruskal Wallis ANOVA χ^2^ = 1.60, df = 2, p = 0.45; total copulations: A = 0, 0–5; B = 0, 0–3; C = 0.5, 0–4; χ^2^ = 1.46, df = 2, p = 0.48). We therefore find no evidence that a nulliparous female benefits from the false oestrus of her relatives, either by having greater choice of males or by copulating more often.

A more subtle benefit might accrue to a young female if a relative's false oestrus served to deflect the persistent advances of young male elephants, which often appear stressful to young females. Mature musth males are known to inhibit musth in younger males [4], [21] and the presence of a guarding musth male deters younger males from harassing inexperienced females [4], [9], [12]. Thus, in addition to their preferred status as mates, the presence of musth males has the additional benefit to a female of reducing potentially stressful copulation attempts of young males. Females might, therefore, simulate oestrus behaviour either to attract older musth males and so protect the naïve, nulliparous females, or to deflect the harassment from the young males onto themselves, away from the young females. However, there was no difference in the number of non-musth males present with the group between the three conditions (median, min-max range of non-musth males present: A = 0, 0–12; B = 0, 0–2; C = 0, 0–3; χ^2^ = 0.61, df = 2, p = 0.74). Similarly, the proportion of attempted copulations by non-musth males did not differ between the three conditions (mean ±sd copulations attempted by non-musth males: A = 0.37±0.5; B = 0.33±0.5; C = 0.35±0.5; χ^2^ = 0.07, df = 2, p = 0.97. Note: mean values are shown here to better illustrate the spread of proportions). Thus, it appears that the act of simulating oestrus behaviour in the presence of a nulliparous female does not serve to directly decrease stress upon a nulliparous female from the attentions of non-musth males.

### Simulation as teaching

The final possibility that we test here is that simulating oestrus in the company of nulliparous females functions as a form of demonstration. That is, do parous females display false oestrus behaviours to coincide with the first occasions that young, nulliparous females come into oestrus, in order to provide information? Demonstration by parous relatives may encourage nulliparous females to direct their oestrus behaviour towards the much larger musth males, rather than running away from them, helping them to learn to attach themselves to males that are able to guard them. We were alerted to this possibility because the long-term AERP researchers who have observed the false oestrus events believe that it does encourage appropriate oestrus behaviour in naïve, nulliparous females [15].

**Figure 2:**
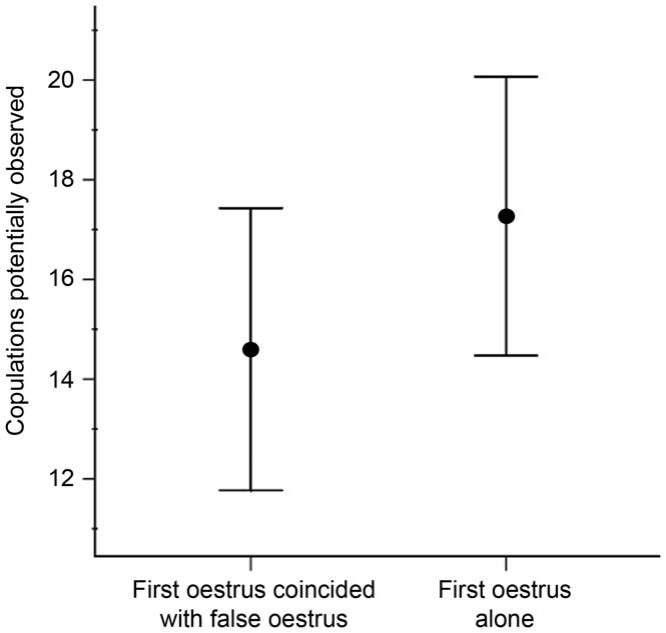
Number of within-family oestrus events observed by nulliparous females. The mean (±2 SE) number of within-family oestrus events that nulliparous females could potentially have observed before their own first oestrus event, according to whether that first oestrus event coincided with a false oestrus event of a family member, or if it occurred alone. The number of oestrus events that were potentially observed is a minimum figure, collated from birth records and the oestrus database.

The 10 nulliparous females whose oestrus coincided with a false event were younger than the average female at first conception; as discussed in the methods section, most nulliparous females do conceive during their first oestrus period (mean age of nulliparous females whose first oestrus coincided with a false oestrus event  = 11.4 years, sd ±1.95; average age at first conception for whole population = 12.9 years [16]; one-sample t-test; t = −2.5, df = 9, p = 0.034). This could indicate their naïve status and therefore their need to learn this information. These ten young females potentially observed an average of at least 14.6 (sd ±4.5) genuine oestrus events of family members before their own first observed oestrus period. We compared these data to the number of oestrus events potentially observed by other nulliparous females in the same families whose first observed oestrus event did not coincide with a false oestrus event of a family member (mean 17.3 events, sd ±8.5) (see figure 2). Young females whose first oestrus did coincide with a false oestrus event had had the opportunity to observe fewer genuine oestrus events by family members than those nulliparous females whose first oestrus did not coincide with any other within-family oestrus event; however, this difference was not significant (t = 1.34, df = 28, p = 0.19, homogeneity of variances not assumed).

The criteria set out by Caro and Hauser [22] in the early 1990's remain the yard-stick for animal teaching, against which all potential cases must be assessed. The key components of their definition are that the ‘teacher’ must only engage in the key behaviour in the presence of the naïve ‘pupil’; there must be a cost to the teacher, or at least no direct benefit; and there must be evidence of learning on the part of the pupil, resulting only from its experience with the teacher.

Firstly, do parous females only demonstrate false oestrus behaviour in the presence of naïve, nulliparous females? Although the distribution of false oestrus is biased toward coincidence with a young, nulliparous female, based on the current data the answer appears to be no: 47% of the 19 false oestrus events observed did not coincide with an oestrus event of a nulliparous female.

Secondly, is there a cost to the teacher? Adopting oestrus behaviours and postures necessarily entails energetic costs of expending energy whilst reducing the time available for feeding. It may also involve further costs: false oestrus behaviour still attracts the attention of males, at least until they can determine its falseness by smelling the urine or vulva of the simulating female. This means that females simulating oestrus will have to deal with the advances of males. Whilst this could have a direct demonstrative effect on the nulliparous females, it is an energetic cost to the simulating female, and may even give her a negative ‘reputation’ among males as a dishonest signaller. Furthermore, the above analyses show there is no direct benefit to the simulating female in terms of reproductive success, so this criterion is met.

Finally, is there any evidence of learning on the part of the pupil? We explored whether the presence of an older, parous female simulating oestrus might result in a greater tendency for nulliparous females immediately to mate with musth males, compared to those females unable to benefit from any such presence. The proportion of mating attempts made by musth: non musth males did not differ according to the presence of other females showing oestrus (see previous section on inclusive fitness), and there was no association between the mate choice of nulliparous females and the presence or absence of an older female showing false oestrus behaviours (Fishers exact: p = 0.74). In the longer term, whether or not a nulliparous female had a false oestrus as a potential model might affect her latency before parturition: but again, we found no such effect. All nulliparous females required a similar number of oestrus cycles before conceiving (median, min-max range months to birth: if first oestrus alone = 24, 20–99 months; if coincident with parous female in oestrus = 25, 21–66; if coincident with parous female showing false oestrus = 28, 21–114; Kruskal Wallis ANOVA χ^2^ = 1.09, p = 0.58). We therefore find no difference in the performance of nulliparous females whether their oestrus event coincided with false oestrus of a parous relative or not.

## Discussion

The analysis presented here confirms that parous female elephants of the Amboseli population do simulate oestrus behaviour, although such simulations are seemingly rare. These false oestrus events occur disproportionately often in the presence of nulliparous female relatives. Genuine oestrus events coincide with the oestrus of another family member only 9% of the time, which is consistent with the brief period of time that females spend in oestrus as opposed to pregnancy or lactational anoestrus: false oestrus events coincide with a nulliparous female's oestrus on at least 53% of occasions. In an attempt to explain the higher-than-expected coincidence of false oestrus with nulliparous female oestrus, we considered the possibility that false oestrus has no functional benefit; that it serves to directly increase reproductive success of the simulating female; or that it increases inclusive fitness of the simulating female, by increasing access for related females to the best males, or by encouraging appropriate behaviours towards the best mates.

False oestrus is quite commonly observed during mammalian pregnancy [23]–[25]. Whilst such hormonal changes may be the proximate cause of false oestrus events here, at least three different abnormal hormonal mechanisms must be invoked to explain the occurrence in senescent females, pregnant females, and females in lactational anoestrus. Moreover, hormonal fluctuations may be sufficient to explain specific instances of false oestrus, but cannot explain the overall pattern of occurrence observed here: that is, the higher-than-expected coincidence of false oestrus with the genuine oestrus events of nulliparous female relatives. Similarly, the occurrence of false oestrus in the presence of young, nulliparous relatives, by females who are already pregnant, senescent, or lactating, is inconsistent with the hypothesis that it serves to directly improve the reproductive success of the parous female. The observed distribution makes it very unlikely that false oestrus has no functional role, or that it functions directly to increase reproductive success.

So does false oestrus benefit inclusive fitness? We found no evidence that nulliparous females experience increased access to musth males, increased copulations, or reduced hassle from young males if their genuine oestrus coincided with a false event. This leaves us with the final possibility that parous females simulate oestrus in order to demonstrate towards which males the oestrus behaviours are most appropriately directed. False oestrus does occur disproportionately often in the presence of nulliparous females who are younger than average at first oestrus, and who may therefore have had less opportunity to observe oestrus behaviours than other nulliparous females in their families. Thus, there is conceivably a need for demonstration. However, only one of Caro and Hauser's three criteria for teaching [22], cost to the teacher, is definitely met by the current data, so it is not possible at present to conclude that false oestrus functions to teach naïve youngsters.

Cases of oestrus simulation analysed here were identified from oestrus and demographic records; at the time, they were sufficient to dupe highly experienced researchers into thinking the females were sexually receptive. However, it is possible that the occurrence of false oestrus by a parous female, coincident with the genuine oestrus of a nulliparous female, is more common than the data here suggest; if the lack of oestrus state is correctly recognized by a researcher, such actions would not be recorded as oestrus behaviour. As noted above, four of the false oestrus events that were not observed to coincide with any other oestrus event *did* occur in the same month as conception by a nulliparous relative. If these false events did actually overlap with the oestrus of these nulliparous females, then only a quarter (5/19) of false oestrus events occurred without any overlap, providing a more convincing response to Caro and Hauser's criterion that the behaviour should be specific to the presence of a naïve pupil. This is a tantalising possibility, and one that encourages us to collect more data on the occurrence of false oestrus events.

The data presented here also do not clearly conform to Caro and Hauser's requirement of a demonstrable learning effect. We found no difference in subsequent mating behaviour between females who had a false oestrus ‘model’ and those that did not. However, the occasional use of demonstration here may have the effect of *correcting* erroneous oestrus behaviour of particularly inept or young nulliparous females, so it is possible that we did not find significant differences on measures of performance precisely *because* simulation does function effectively as teaching. Clear demonstration of a specific learning effect necessarily requires experimental manipulation of the behaviour in question [26]; using experiments, teaching has now been demonstrated in widely divergent species (ants, meerkats, and pied babblers) [27]–[30]. There is also suggestive evidence of teaching in other species such as raptors, cheetahs, certain species of primates, and cetaceans [31]–[32], where experimental manipulation has not been conducted, and which therefore do not fully satisfy the learning criterion.

This analysis of 28-years of longitudinal data on the demography and oestrus behaviour of African elephants provides the first step in understanding why elephants sometimes simulate oestrus behaviours. From these data, we have demonstrated that elephants do not simulate oestrus randomly, but target their occasional efforts at certain nulliparous female relatives, possibly individuals they assess as being particularly young or naive. Further data is required to confirm or reject the hypothesis that this behaviour functions to teach the young, naïve females, but we suggest that it remains the only viable possibility based on the current analyses. If it can be demonstrated, by targeted data collection, that false oestrus is directed towards nulliparous females who were performing below par before the simulation, then we would be as close as possible without experimental manipulation to meeting Caro and Hauser's criteria for animal teaching.

## Supporting Information

Table S1 Data table to accompany Document S1.
(0.03 MB DOC)

Document S1 Female mate choices. Analysis showing that nulliparous female elephants do not direct their oestrous behaviour appropriately towards musth males, unlike parous females.
(0.02 MB DOC)
