# Supplementary material for: Why Do African Elephants (Loxodonta africana) Simulate Oestrus? An Analysis of Longitudinal Data
Source: PLoS One. 2010 Apr 7;5(4):e10052. doi: 10.1371/journal.pone.0010052 (PMC2850927; doi:10.1371/journal.pone.0010052)
Supplement: Table S1 — Data table to accompany Document S1. (0.03 MB DOC) [file pone.0010052.s001.doc]

Matings or attempted matings where the oestrous female initially ran away.

|  | Pursuing male | |
| --- | --- | --- |
|  | Musth male | Non-musth male |
| Parous female | 3 | 73 |
| Nulliparous female | 5 | 11 |
