# Supplementary material for: Why Do African Elephants (Loxodonta africana) Simulate Oestrus? An Analysis of Longitudinal Data
Source: PLoS One. 2010 Apr 7;5(4):e10052. doi: 10.1371/journal.pone.0010052 (PMC2850927; doi:10.1371/journal.pone.0010052)
Supplement: Document S1 — Female mate choices. Analysis showing that nulliparous female elephants do not direct their oestrous behaviour appropriately towards musth males, unlike parous females. (0.02 MB DOC) [file pone.0010052.s002.doc]

**Female mate choices**

Nulliparous females often do not direct their behaviours appropriately when they first come into oestrus. Nulliparous females ran away from musth males more often than expected, as shown in Table S1; in 16 observations of a nulliparous oestrous female running away from an interested male, 31% of cases (n=5) described the nulliparous female running away from a musth male. This is in contrast to parous oestrous females, who were only observed to run from musth males in 4% (n=3) of the total 76 observations of them running away from an interested male. (Note, not all of these interactions resulted in a copulation). This distribution is different to that expected by chance (Fisher’s exact p=0.004).

When considering all interactions that did result in an observed copulation between oestrous females and musth males (parous females and musth males, n=93; nulliparous females and musth males, n=81), nulliparous females in oestrus were more likely to run away from the musth male than was an experienced, parous oestrous female (nulliparous 5 out of 81 = 6%; parous 2 out of 93 = 2%; binomial test: nulliparous females ran away from musth males = 5, nulliparous females stood for musth males = 76, test proportion = 0.02, p<0.001).
